# An injection and mixing element for delivery and monitoring of inhaled nitric oxide

**DOI:** 10.1186/s12938-016-0227-5

**Published:** 2016-08-30

**Authors:** Andrew R. Martin, Chris Jackson, Samuel Fromont, Chloe Pont, Ira M. Katz, Georges Caillobotte

**Affiliations:** 1 Department of Mechanical Engineering, University of Alberta, 10-324 Donadeo Innovation Centre for Engineering, Edmonton, AB T6G 1H9 Canada; 2 Virginia Commonwealth University, Richmond, VA 23284 USA; 3 Medical R&D, Air Liquide Santé International, Centre de Recherche Paris-Saclay, 1 Chemin de la Porte des Loges, B.P. 126, 78354 Les Loges-en-Josas, France; 4 Department of Mechanical Engineering, Lafayette College, Easton, PA 18042 USA

## Abstract

**Background:**

Inhaled nitric oxide (NO) is a selective pulmonary vasodilator used primarily in the critical care setting for patients concurrently supported by invasive or noninvasive positive pressure ventilation. NO delivery devices interface with ventilator breathing circuits to inject NO in proportion with the flow of air/oxygen through the circuit, in order to maintain a constant, target concentration of inhaled NO.

**Methods:**

In the present article, a NO injection and mixing element is presented. The device borrows from the design of static elements to promote rapid mixing of injected NO-containing gas with breathing circuit gases. Bench experiments are reported to demonstrate the improved mixing afforded by the injection and mixing element, as compared with conventional breathing circuit adapters, for NO injection into breathing circuits. Computational fluid dynamics simulations are also presented to illustrate mixing patterns and nitrogen dioxide production within the element.

**Results:**

Over the range of air flow rates and target NO concentrations investigated, mixing length, defined as the downstream distance required for NO concentration to reach within ±5 % of the target concentration, was as high as 47 cm for the conventional breathing circuit adapters, but did not exceed 7.8 cm for the injection and mixing element.

**Conclusion:**

The injection and mixing element has potential to improve ease of use, compatibility and safety of inhaled NO administration with mechanical ventilators and gas delivery devices.

## Background

Inhaled nitric oxide (NO) is known to act as a selective pulmonary vasodilator [1, 2], and is currently indicated for use in the treatment of hypoxic respiratory failure of the term and near-term newborn [3]. Additional use in improving oxygenation in adult patients with acute lung injury or acute respiratory distress syndrome [4, 5], and in alleviating pulmonary hypertension in both adults and children post cardiac surgery [6, 7], has been well-documented. The vast majority of patients receiving inhaled NO do so in the critical care setting, and are concurrently supported by invasive or noninvasive positive pressure ventilation. As such, devices developed to administer NO to patients must interface with the ventilator breathing circuit and coordinate with the breathing cycle. Current marketed NO delivery devices do so by injecting source NO-containing nitrogen (800 parts per million, ppm, NO in balance nitrogen, N_2_, in North America; 225–1000 ppm NO in N_2_ in Europe) into the inspiratory limb of the breathing circuit. The injection flow rate is adjusted in proportion to the flow rate of air/oxygen in the circuit so as to maintain a constant, target NO concentration in the inhaled gas mixture. Fittingly, dosing recommendations have been established based on the NO concentration in inhaled gas [4, 8].

An important function of NO delivery devices is to sample the inhaled gas mixture downstream from the point of NO injection so as to establish whether or not target NO concentrations are met [9–11]. Sampled gas is also monitored for nitrogen dioxide (NO_2_), a toxic reaction product when NO is in the presence of oxygen. Physical spacing between the injection and sampling points is required so that injected NO adequately mixes with breathing circuit gases before being sampled [12]. In practice, the injection point is positioned close to the ventilator and the sampling point positioned close to the patient, so that transit of gases through the inspiratory limb of the breathing circuit provides ample mixing time. Several drawbacks are associated with this practice. First, while increased NO residence time in breathing circuits is beneficial for gas mixing, production of NO_2_ increases with increased residence time as well. A recent bench investigation of NO delivery through neonatal noninvasive respiratory support devices measured potentially dangerous NO_2_ concentrations (>2 ppm) in certain worst-case scenarios related to extended gas residence times in breathing circuits [13]. Second, for newer, noninvasive forms of respiratory support, such as high flow nasal cannula therapy [14, 15], gas delivery conduits may lack sufficient internal volume to ensure mixed samples, so that modifications are required at the device level to enable compatibility with NO delivery. Finally, given the wide range of invasive and noninvasive forms of respiratory support currently available in intensive care units, there exists potential for human error in placing NO injection and sampling connections at appropriate positions within a diverse range of breathing circuits and gas delivery apparatus.

It is therefore desirable to move towards NO injection and sampling apparatuses capable of safe and effective operation with limited specific restrictions on their positioning within breathing circuits. Such apparatuses would serve the dual purpose of ensuring ease of setup and compatibility with a wide range of respiratory support devices, while permitting NO injection to occur closer to the patient, thereby reducing NO residence time in the circuit and associated NO_2_ production. In the present article, a NO injection and mixing element is presented. The device borrows from the design of traditional static elements to promote rapid mixing of injected NO-containing gas with breathing circuit gases. Bench experiments are presented to demonstrate the improved mixing afforded by the injection and mixing element as compared with injection through two commercially-available breathing circuit adapters used for NO injection with marketed NO delivery devices. CFD simulations are also presented to illustrate mixing patterns and NO_2_ production within the element.

## Methods

### Experimental measurements

**Fig. 1:**
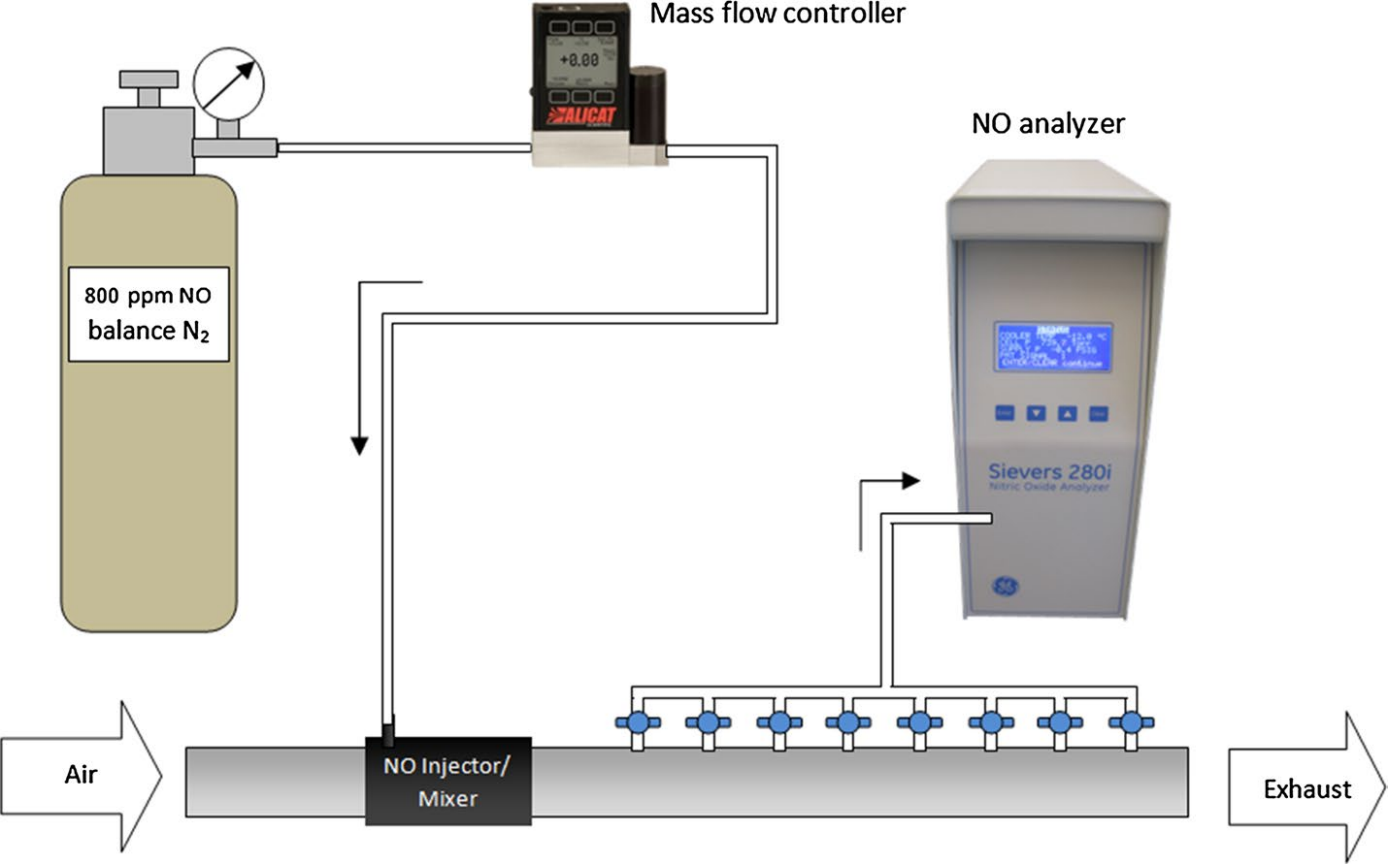
Schematic of experimental apparatus used for measuring nitric oxide (NO) concentration downstream from injection site. Note that the actual number of sampling ports was 16

Experiments were conducted to determine the downstream distances required to mix injected NO-containing gas (800 ppm NO in balance N_2_; American Air Liquide, USA) into steady flows of air within standard 22 mm breathing circuit tubing and connections. Air flow rate was set using a rotameter (FME Series; Western Medica, USA) for flow rates between 2 and 10 standard liters per minute (l/min) and a second rotameter (King Instrument Company, USA) for 40 l/min flow rates. A 2 m length of straightened breathing circuit tubing was positioned upstream from the point of NO injection. Adapters used for NO injection were followed by a series of 16 respiratory gas sampling ports (22M–22F with 10 M Swivel Elbow; Intersurgical, UK), as shown schematically in Fig. 1. The flow rate of injected NO-containing gas was set using a mass flow controller (MCS-2SLPM-D/5 M; Alicat Scientific, USA) and was adjusted according to the air flow rate to achieve final NO concentrations in the mixed gas of 10, 20 and 40 ppm NO. As depicted in Fig. 1, the 16 sampling points were connected via stopcocks such that gas was sampled from a single sampling point at a time to a Sievers 280i NO analyzer (General Electric; USA). The sampling flow rate was held constant throughout experiments at 200 ml/min. The NO analyzer was connected via serial communication to a personal computer, and a LabView (National Instruments, USA) based virtual instrument was written for data acquisition.

For a given experimental run, steady flow rates of air and of NO-containing gas were set, and then NO concentration at each sampling point was measured. A sampling interval of 5 s was used at each point, and the average NO concentration over the interval was calculated and recorded. Experiments were repeated in triplicate, with rotameter and mass flow controller set points reset between repetitions. Measured NO concentrations are reported below as the average ± standard deviation between repetitions. For two commercially-available breathing circuit adapters used for NO injection (described below), concentration measurements were made both with sampling points at the same angular position as the NO injection (i.e. at the top of the main flow conduit, as depicted in Fig. 1) and rotated 180° from the NO injection point (i.e. at the bottom of the main flow conduit). As no significant differences were noted between top and bottom sampling in downstream distance required for NO concentration to reach within ±5 % of the final target concentration, further experiments were conducted only with sampling points positioned at the top of the flow conduit.

**Fig. 2:**
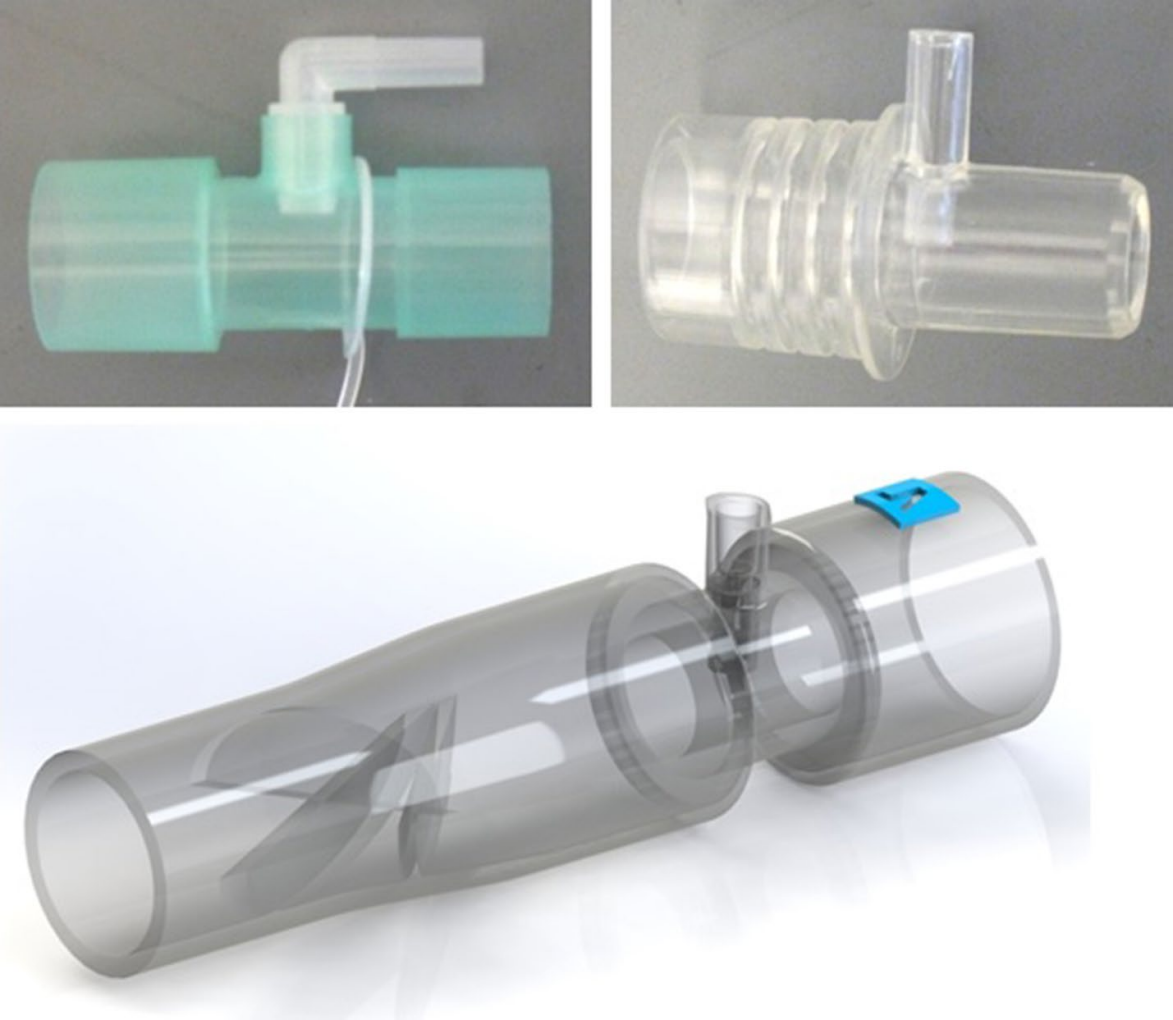
Respiratory gas sampling adapters used for nitric oxide injection (*top left* Adapter A; *top right* Adapter B) along with the injection and mixing element (*bottom*). Air flow through the adapters and mixing element was from *right* to *left*

**Fig. 3:**
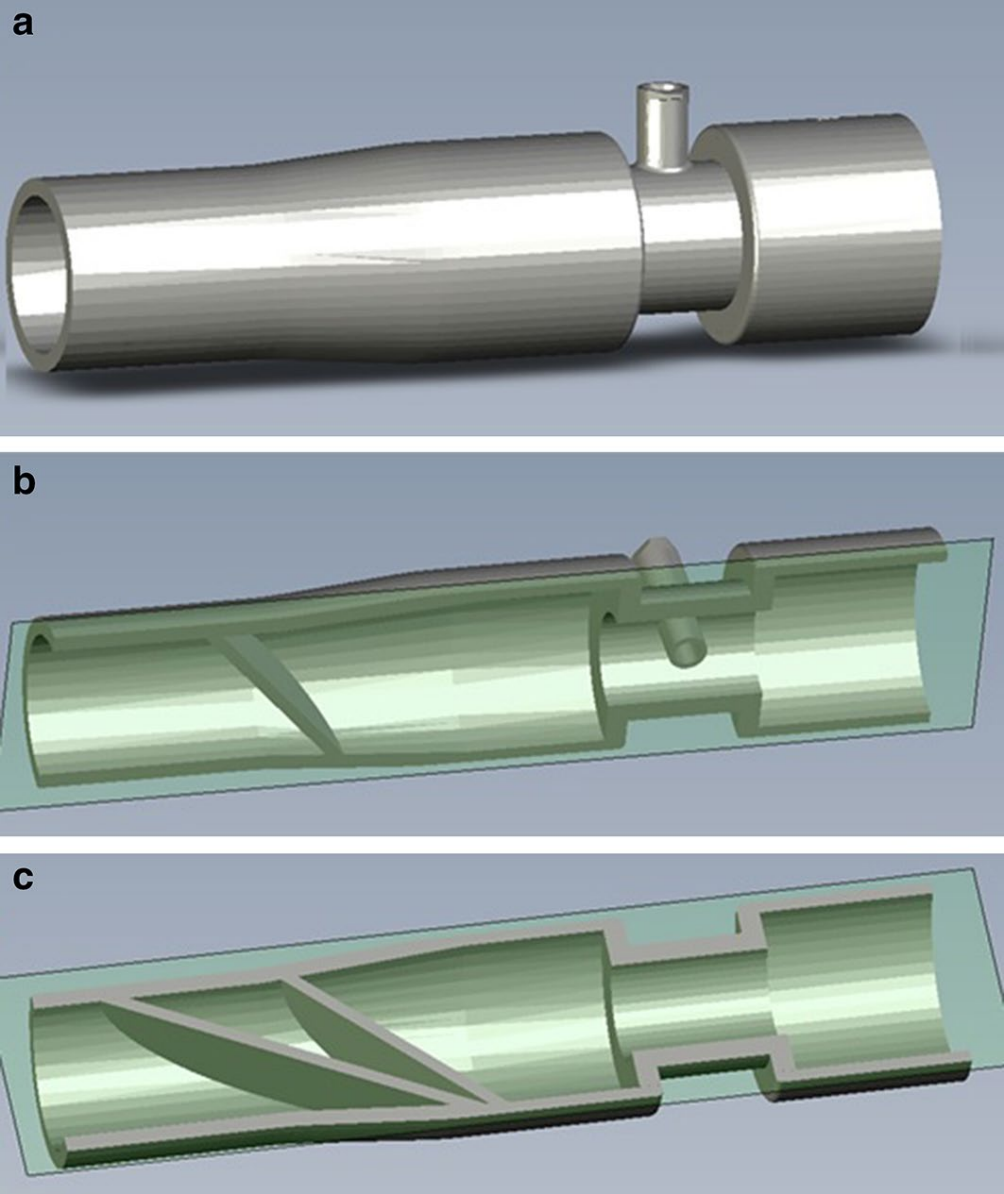
**a** Computer-aided design (CAD) rendering of the injection and mixing element, along with views of **b** the *top half* and **c** the *bottom half* of the element to expose the internal geometry

As noted above, two commercially-available breathing circuit adapters were evaluated for NO injection. Both adapters are respiratory gas sampling ports that have been repurposed as NO injection ports for use with marketed NO delivery devices. These are shown in Fig. 2, and will be referred to below as Adapter A (22M–22F with 10 M Swivel Elbow; Intersurgical, UK) and Adapter B (Medical Gas Sampling Straight Connector; Smiths, UK). Downstream distances required to achieve final NO concentrations for the two adapters were compared to those for the NO injection and mixing element, depicted in Figs. 2 and 3. A prototype of the injection and mixing element was designed in Solidworks (Dassault Systemes, France) and built for testing in R5 Gray resin using an Ultra 3D printer (EnvisionTEC, USA), with layer thickness of 50 µm and in plane resolution of 139 µm. Two versions of the NO injection and mixing element were built and tested: the first, as shown in Figs. 2 and 3, included a sudden constriction in internal diameter from 22 to 12 mm in the position of NO injection, while the second included no such constriction, such that the inner diameter remained at a constant 22 mm from the inlet through the injection point.

In addition to NO concentration measurements described above, the pressure drop across the adapters, and each version of the injection and mixing element, was evaluated using a digital manometer (HD755 ± 0.5 psi range Differential Pressure Manometer; Extech Instruments, USA) at the maximum air flow rate studied, 40 l/min.

### Computational fluid dynamics simulations

Steady state CFD simulations were performed using the finite volume solver FLUENT (ANSYS; USA) for Adapter A and for the injection and mixing element. A laminar model of the Navier–Stokes (NS) equations and a transitional turbulence model were used for Adapter A and for the injection and mixing element, respectively. Second-order-accurate discretization schemes were used for all terms. Pressure–velocity coupling was achieved using the SIMPLE algorithm, and the transitional *k*-*kl*-*ω* model, a three-equation eddy-viscosity model for laminar and turbulent kinetic energies (*k* and *kl*, respectively) as well as inverse turbulent time scale (*ω*), was incorporated. The transitional model is based on two transport equations, one for intermittency and one for the transition onset criteria in terms of momentum thickness Reynolds number. The transport equations are intended for the implementation of correlation-based models into general-purpose CFD methods [16]. The theoretical framework for the CFD methods, including the SIMPLE algorithm and correlation constants for the turbulence model, is provided in the FLUENT Theory Guide [17]. The boundary conditions included no-slip and no-penetration at the walls; parabolic laminar velocity profiles (using a user defined function) for the air flow and NO injection flow, and the primary outlet boundary was given classic outflow conditions forcing downstream velocity derivatives to be zero. The CFD simulations included 11 sampling points, each drawing prescribed flow rates of 18.2 ml/min, for a total flow of 200 ml/min equal to the flow rate through a single port during the physical experiment. A mesh refinement study using grids with 2, 4, and over 9 million cells was performed. The grid with 4 million cells used for this study converged to within 0.9 % of the finest grid for flow variables.

The convection, diffusion, and chemical reaction of gaseous species was solved according to with the following equation:

1 $$ \frac{\partial }{\partial t}\left( {\rho Y_{i} } \right) + \nabla \cdot \left( {\rho \vec{v}Y_{i} } \right) = - \nabla \cdot \overrightarrow {{J_{i} }} + R_{i} $$

where *Y*_*i*_ is the mass fraction of NO, NO_2_, O_2_, or N_2_; *ρ* is the density of the gas mixture; $\vec{v}$ is the fluid velocity; *R*_*i*_ is the net rate of production of species *i* by chemical reaction; and the diffusion flux of species *i* is expressed as:

2 $$ \overrightarrow {{J_{i} }} = - \mathop \sum \limits_{j = 1}^{N - 1} \rho D_{ij} \nabla Y_{j} - D_{T,i} \frac{\nabla T}{T} $$

where *N* = *4* is the number of species, *D*_*ij*_ is the binary mass diffusion coefficient, computed according to the Chapman-Enskog formula; *D*_*T,i*_ is the thermal diffusion coefficient, and *T* is temperature.

The CFD simulations additionally included production of NO_2_, based on the chemical reaction:

3 $$ 2\rm{NO} + \rm{O_{2}} = 2\rm{NO_{2}} $$

The rate of reaction was based on the component concentrations and the constant *k*:

4 $$ \frac{{{\text{d}}[\rm{NO_{2}} ]}}{{{\text{d}}t}} = - \frac{{{\text{d}}[\rm{NO]}}}{{{\text{d}}t}} = 2k \left[ \rm{{NO}} \right]^{2} \left[ \rm{{O_{2} }} \right] $$

where *k* was determined using the Arrhenius expression [18]:

5 $$ k = 1200e^{530/T} $$

where *T* is the temperature in Kelvin, and *k* has units of L^2^/mol^2^/s.

## Results

### Experimental measurements

**Fig. 4:**
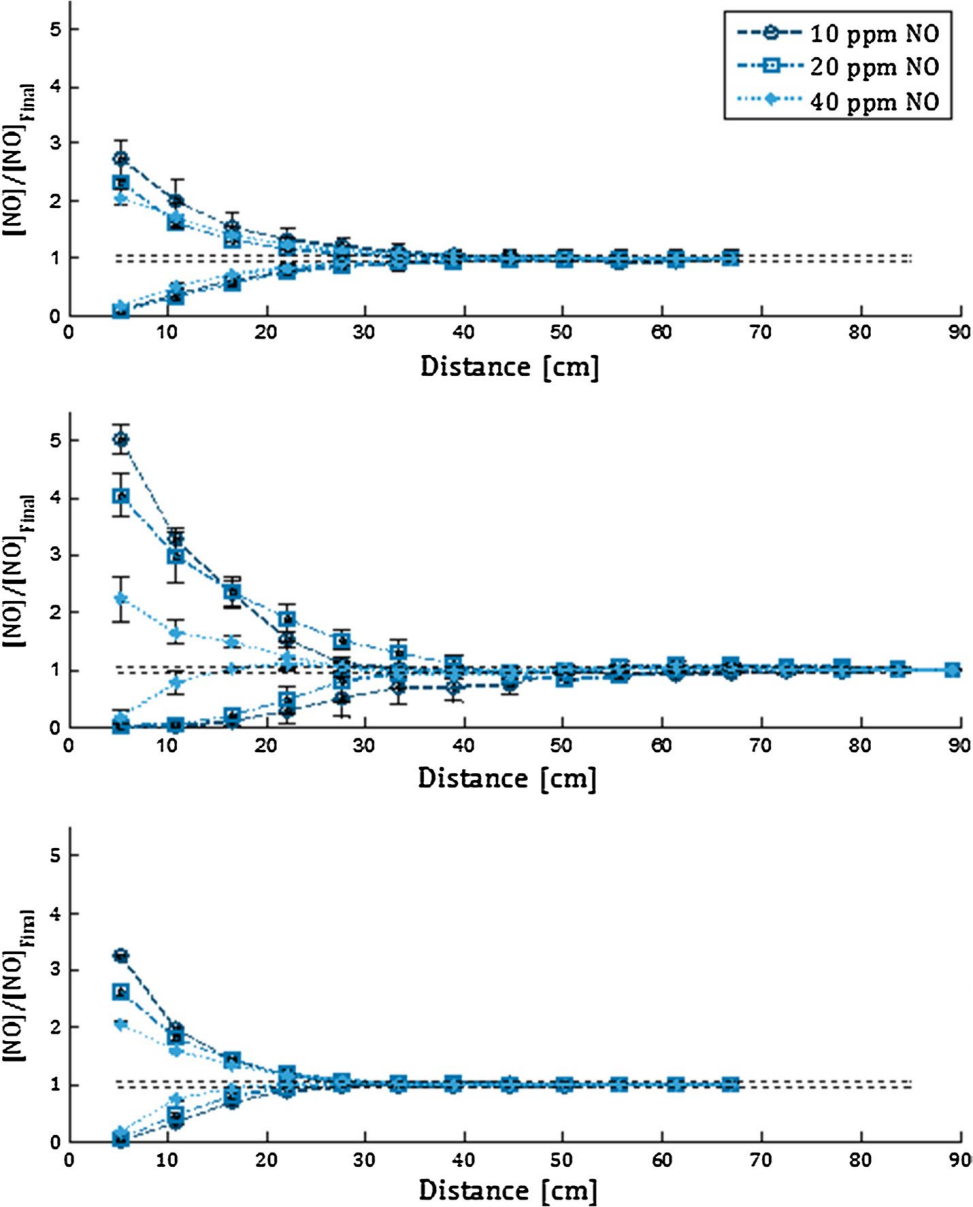
Normalized NO concentration is plotted against the distance downstream from the point of NO injection using Adapter A, for air flow rates of 2 l/min (*top*), 10 l/min (*middle*), and 40 l/min (*bottom*)

**Fig. 5:**
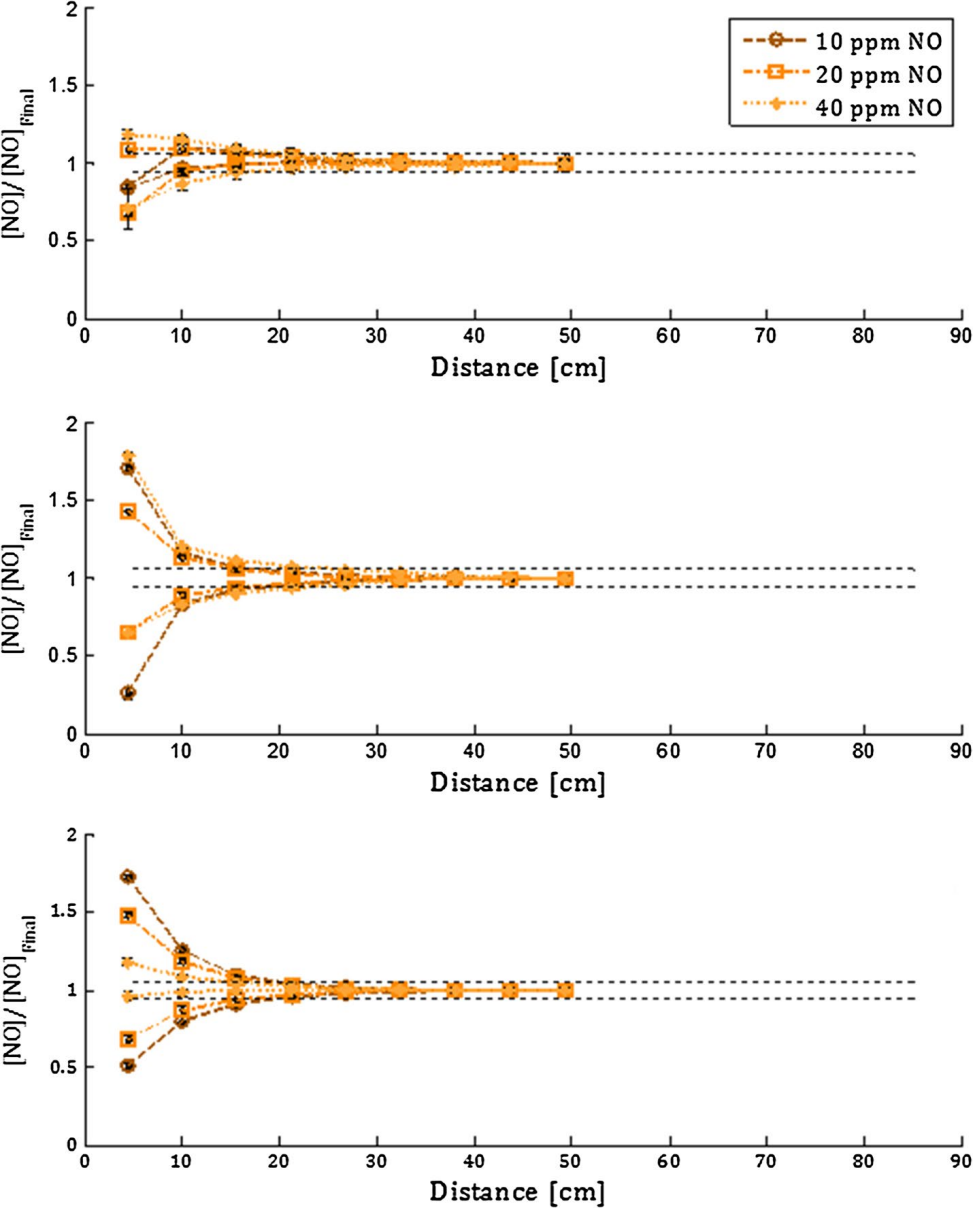
Normalized NO concentration is plotted against the distance downstream from the point of NO injection using Adapter B, for air flow rates of 2 l/min (*top*), 10 l/min (*middle*), and 40 l/min (*bottom*)

**Fig. 6:**
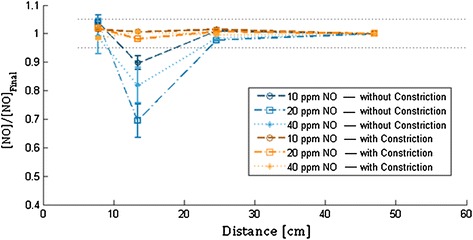
Normalized NO concentration is plotted against the distance downstream from the point of NO injection using the injection and mixing element designs with and without constriction in the region of injection. The air flow rate is 10 l/min

Figures 4 and 5 display NO concentrations measured at sampling points positioned at varying distance downstream from the point of NO injection for Adapter A and B, respectively. These measurements are displayed for the two versions of the injection and mixing element (with and without constriction) in Fig. 6, for air flow rate of 10 l/min. For the 2 and 40 l/min air flow rates, both versions of the injection and mixing element yielded NO concentration within ±5 % of the final target concentration at all sampling locations for all three target concentrations. The mixing length was defined as the downstream distance required for NO concentration to reach within ±5 % of the final target concentration, and is summarized for the two adapters and the two versions of the injection and mixing element in Table 1. The pressure drop at 40 l/min measured across each adapter or element is also reported in Table 1.

**Table 1:** Mixing length and pressure drop for injection apparatus

Description	Mixing length^a^ (cm)	Pressure drop^f^ (Pa)
Adapter A	47 ± 7^b^	2 ± 0
Adapter B	27 ± 1^c^	44 ± 3
Custom w/out constriction	23 ± 1^d^	33 ± 2
Custom w/constriction	<7.8^e^	57 ± 5

Values are mean ± standard deviation (n = 3)

^a^The worst case mixing distances of the three air flow rates and three target NO concentrations are shown

^b^Worst case mixing occurred for 10 l/min and 10 ppm

^c^Worst case mixing occurred for 10 l/min and 10 ppm

^d^Worst case mixing occurred for 10 l/min and 20 ppm

^e^Indicates that the NO concentration was within 5 % of the final target concentration by the first sampling point for all repeated experiments at each combination of air flow rate and target NO concentrion. The distance to the first sampling point is indicated

^f^Pressure drop was evaluated for air flow at 40 l/min

### Computational fluid dynamics simulations

**Fig. 7:**
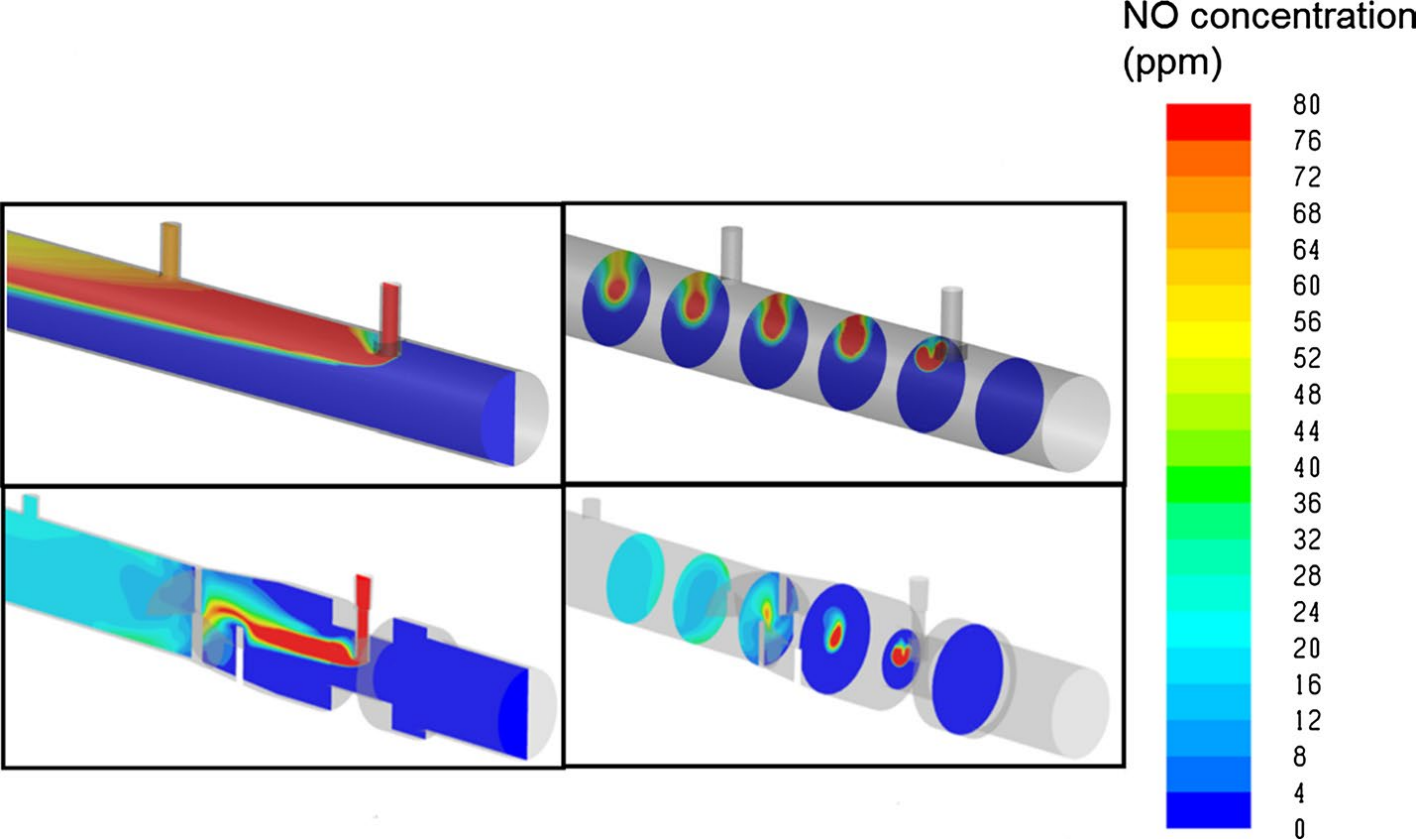
Simulated NO concentrations are displayed for air flow rate of 10 l/min and target NO concentration of 20 ppm for Adapter A (*top row*) and for the injection and mixing element, with constriction (*bottom row*)

**Fig. 8:**
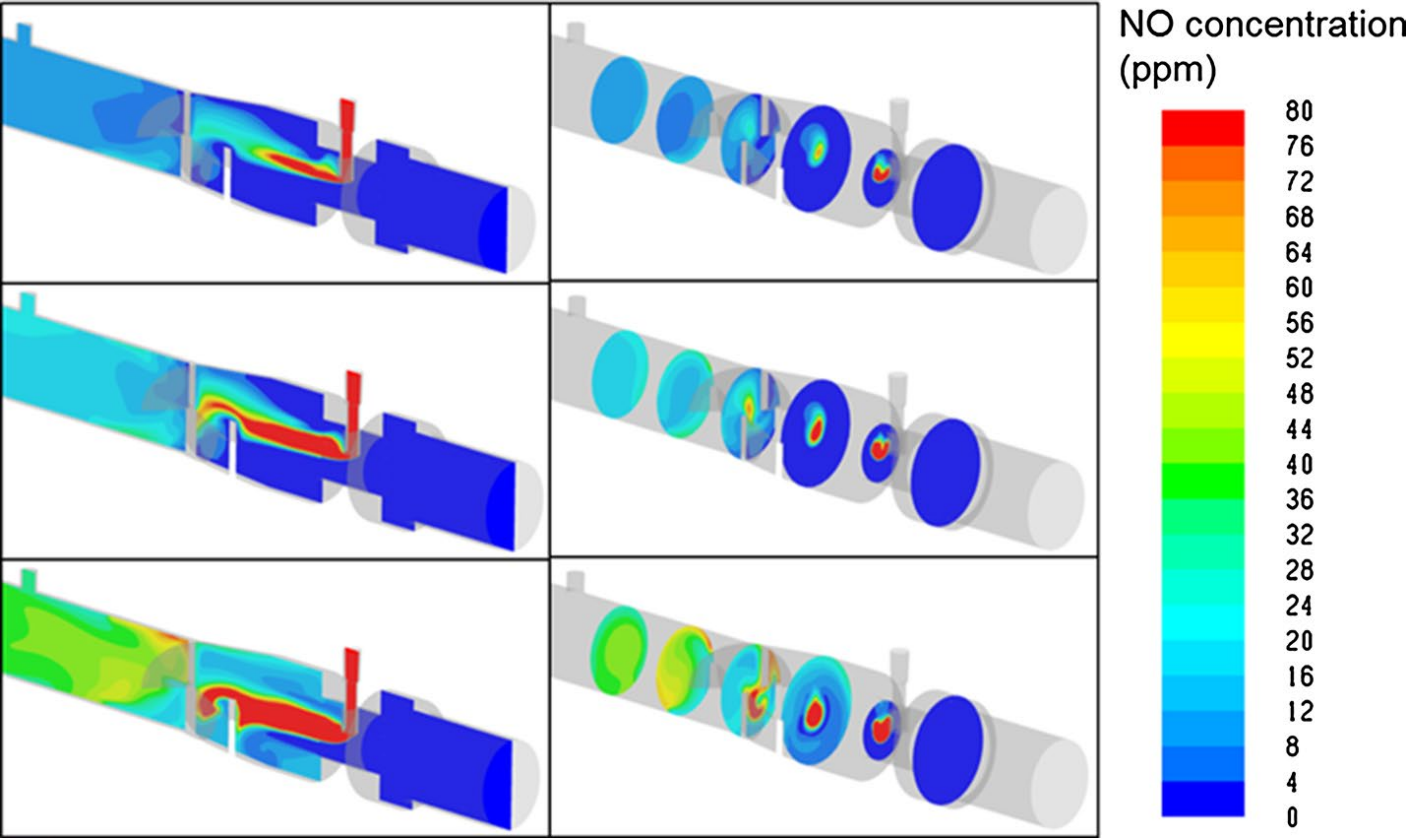
Simulated NO concentrations are displayed for air flow rate of 10 l/min through the injection and mixing element, with constriction for target NO concentration of 10 ppm (*top row*), 20 ppm (*middle row*), and 40 ppm (*bottom row*)

**Fig. 9:**
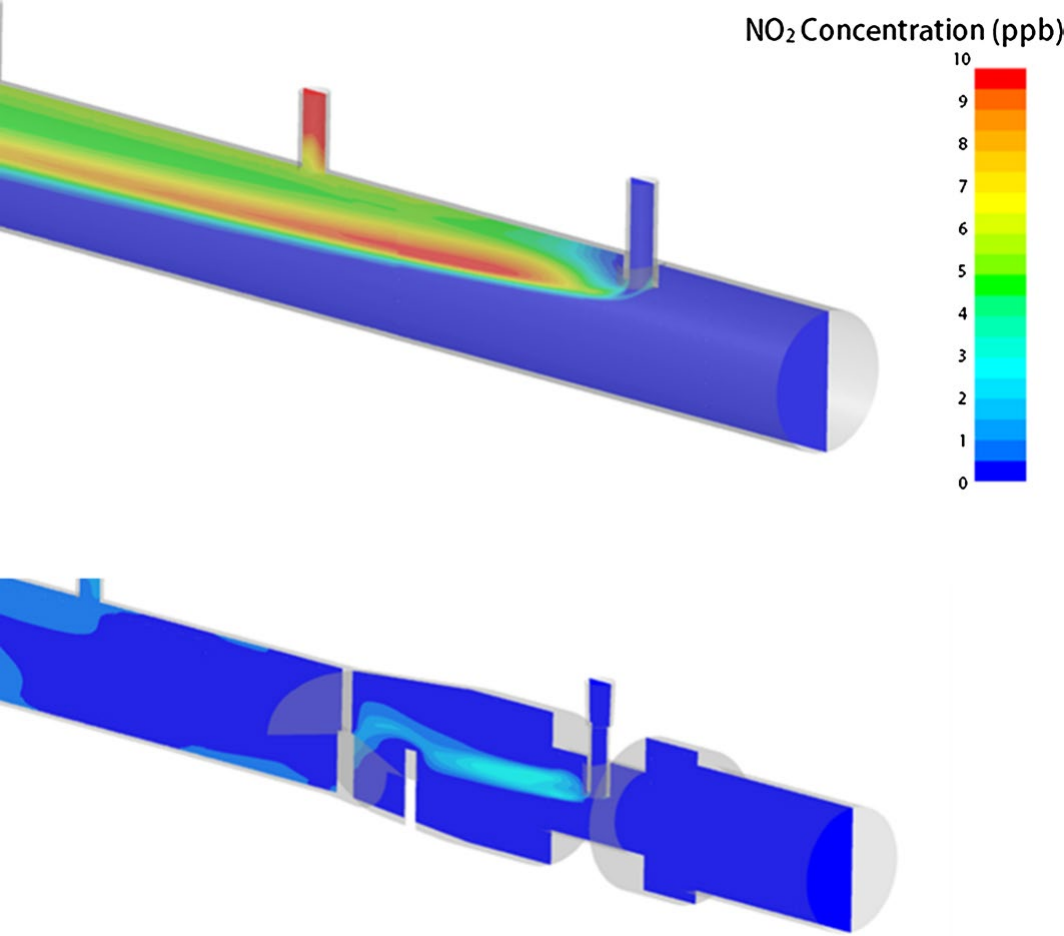
Simulated NO_2_ concentrations are displayed for air flow rate of 10 l/min and target NO concentration of 20 ppm for Adapter A (*top*) and the injection and mixing element, with constriction (*bottom*)

CFD simulations of NO concentration downstream from injection points were qualitatively similar to the experimental measurements, and permit visualization of the mixing process inside the adapters and the injection and mixing element. For example, Fig. 7 compares simulated NO concentrations within Adapter A and the injection and mixing element (with constriction) for the case of 10 l/min air flow and a target 20 ppm NO concentration. Similarly, Fig. 8 displays NO concentrations for the injection and mixing element (with constriction) for 10 l/min air flow and for target NO concentration of 10, 20, and 40 ppm. Simulated NO_2_ concentrations for 10 l/min air flow and target NO concentration of 20 ppm are shown in Fig. 9 for both Adapter A and for the injection and mixing element (with constriction). For these simulations, the area-weighted average NO_2_ concentration at the outlet of the CFD domain was 4.3 ppb for Adapter A and 2.5 ppb for the injection and mixing element.

## Discussion

The present work was conducted in order to explore the design of a novel NO injection and mixing element for use in ventilator breathing circuits and other breathing gas delivery conduits. Experimental results indicate that the downstream distance required to achieve complete mixing between injected NO-containing gas and breathing gas (air, in the present experiments) was greatly reduced when using the injection and mixing element in place of conventional breathing circuit adapters. Indeed, for the final injection and mixing element design, which included a cross-sectional area constriction at the location of NO injection, measured NO concentrations were within ±5 % of final target concentrations at the sampling point positioned closest to the element (a distance of 7.8 cm from the point of NO injection) in all cases studied. In contrast, for Adapter A, a distance of up to 47 ± 7 cm was required downstream of the NO injection point before measured NO concentrations were within ±5 % of final target concentrations. This latter result highlights the importance of instructions provided with current delivery devices for positioning NO injection and sampling points at opposing ends of inspiratory tubing. Without sufficient mixing between NO injection and sampling, monitored NO and NO_2_ concentrations will be inaccurate.

CFD simulations were conducted to explore and visualize NO concentrations within the internal volumes of Adapter A and the injection and mixing element (with constriction), selected as the worst and best performing devices, respectively, in the experimental study. For the injection and mixing element, simulations indicated enhanced mixing between the NO-containing gas stream and air stream resulting from separation and recombination of the gas mixture along different flow paths through the three angled fins positioned immediately downstream from the NO injection point. This is similar to the mixing process through traditional static mixing elements used in a variety of engineering fields, which contain multiple baffles in series, each similar to the three-fin design used at present. For use in breathing circuits, low resistance to flow is critical; therefore, a single (non-repeated) mixing element is preferred.

A small additional improvement to mixing afforded by the custom injection and mixing element presented herein resulted from the inclusion of a cross-sectional area constriction at the location of NO injection. Without this constriction, the inner diameter of the element was 22 mm through the location of NO injection. With the constriction included, the inner diameter was reduced to 12 mm for a short length of 13 mm centered on the location of NO injection. It is hypothesized that inclusion of the constriction enhanced mixing by increasing local air speeds at the point of injection and immediately upstream from the three mixing fins.

The pressure drop measured across the injection and mixing element with cross-sectional area constriction at the location of NO injection, 57 Pa (or 0.58 cm H_2_O) at 40 l/min, was greater than that for the injection and mixing element with no constriction, 33 Pa (0.34 cm H_2_O) at 40 l/min. However, both were well below pressure levels commonly targeted during pressure support or pressure control mechanical ventilation (which may range from ~10 to 40 cm H_2_O), such that positioning the element in-line in the inspiratory limb of a ventilator breathing circuit would have minimal effect on pressures present in patient airways. Indeed, the pressure drop measured through commercial Adapter B, with a minimum inner diameter similar to that of the injection and mixing element with constriction, was of a similar magnitude, 44 Pa (0.45 cm H_2_O). Adapter B is widely used in ventilator breathing circuits, with no reported concerns over imposed resistance to ventilator flows.

CFD simulations also estimated NO_2_ levels produced in Adapter A and the injection and mixing element through the reaction of NO with oxygen. In both cases, NO_2_ concentration remained well below 1 ppm, a level associated with the onset of deleterious health effects [11]. Despite these low levels for the cases simulated, it is noted that NO_2_ production was reduced using the injection and mixing element. This is attributed to a reduced time during which high concentrations of NO were in the presence of oxygen, i.e. during incomplete mixing; the rate of NO_2_ production is proportional with the square of NO concentration (Eq. 4, above). Although increasing oxygen concentration would yield a proportional increase to the rate of NO_2_ production, for the constant flow rate simulations performed, NO_2_ levels would remain well below 1 ppm. In contrast, during cyclic flow conditions, as occurs in breathing circuits with low or no bias flow, the time available for reaction of NO with oxygen between inspiratory cycles is greatly increased. Whether or not improved initial mixing of NO-containing and oxygen-containing gas streams can reduce NO_2_ formation in breathing circuits under cyclic flow conditions remains to be studied. In practice, additional and potentially more significant reduction in NO_2_ production would be afforded by positioning the injection and mixing element close to the patient, thereby reducing NO residence time in the breathing circuit. Such a reduction may be important when considering options to guard against NO_2_ exposure [13, 19].

The present study demonstrates improved mixing between injected NO-containing gas and air flow through breathing circuits for the injection and mixing element as compared with Adapters A and B. That said, certain limitations can be discussed. CFD simulations reported herein served to illustrate notably different spatial mixing patterns between Adapter A, which performed worst in the experimental study, and the injection and mixing element (with constriction), which performed best in the experimental study. Detailed comparison between experimental measurement and CFD simulation of NO concentration in gases sampled downstream from the injection point is not presented. While qualitatively similar NO concentrations were observed, minor differences were present between experimental data and CFD simulations. The goal of the present work was not to validate CFD simulations for quantitative predictions, but rather to use CFD as a tool for visualization of mixing phenomenon, so as to supplement our experimental results. Further, the present experiments were restricted to constant flows of air and NO-containing gas. In practice, for many modern mechanical ventilators, gas flow through the inspiratory limb of a breathing circuit will vary during the breathing cycle. The flow rate of injected NO-containing gas is therefore adjusted in proportion to the breathing circuit gas flow, in an attempt to maintain constant NO concentration in the mixed gas downstream. Use of the injection and mixing element with time varying flows of air, or air/oxygen mixtures, and NO-containing gas remains to be explored. In this case, NO concentrations measured downstream from NO injection will again depend on mixing that occurs between the injection and sampling points, but also on any appreciable time lag between variation in the flow rate of air/oxygen and proportional adjustment of the flow of injected NO-containing gas. Accordingly, the present results are most readily applicable to the subclass of ventilators and breathing support devices which maintain a constant flow rate of gases supplied to the patient. This includes, for example, devices used to administer high flow nasal cannula therapy and constant-flow transport ventilators.

## Conclusions

In summary, a NO injection and mixing element was described that permits rapid mixing of injected NO-containing gas with breathing circuit gases. Bench experiments demonstrated improved mixing afforded by the injection and mixing element compared with conventional breathing circuit adapters. Computational fluid dynamics (CFD) simulations illustrated mixing patterns and NO_2_ production within the element. The injection and mixing element has potential to improve ease of use, compatibility, and safety of inhaled NO administration with mechanical ventilators and other gas delivery devices.

## Abbreviations

CFD: computational fluid dynamics
N_2_: nitrogen
NO: nitric oxide
NO_2_: nitrogen dioxide
NS: Navier–Stokes (equations)
O_2_: oxygen
ppm: parts per million
